# Changes in insecticide resistance and host range performance of planthoppers artificially selected to feed on resistant rice

**DOI:** 10.1016/j.cropro.2019.104963

**Published:** 2020-01

**Authors:** Finbarr G. Horgan, Charle Patrick F. Garcia, Fay Haverkort, Peter W. de Jong, Jedeliza B. Ferrater

**Affiliations:** a EcoLaVerna Integral Restoration Ecology, Bridestown, Kildinan, Co. Cork, Ireland; b University of Technology Sydney, 15 Broadway, Ultimo, Sydney, NSW, 2007, Australia; c International Rice Research Institute, DAPO Box 7777, Metro Manila, Philippines; d Laboratory of Entomology, Wageningen University and Research, P.O. Box 8031, 6700, EH Wageningen, Netherlands

**Keywords:** Host plant resistance, *Bph3(t)* gene, *BPH32*, brown planthopper, Fipronil, Imidacloprid, Insecticide resistance, *Nilaparvata lugens*

## Abstract

Host plant resistance has received considerable attention for the management of insect herbivores on crop plants. However, resistance is threatened by the rapid adaptation of target herbivores towards virulence (the ability to survive, develop and damage a host with major resistance genes). This study examines the potential costs and benefits of adaptation for virulence in herbivores. We continuously reared planthoppers, *Nilaparvata lugens*, on two susceptible and three resistant rice, *Oryza sativa*, varieties for 20 + generations. We then assessed the performance of selected planthoppers across a range of rice lines with distinct resistance genes. We found that planthoppers with long-term exposure to resistant hosts (particularly IR62 with the *Bph3(t)* and *BPH32* gene loci, and PTB33 with the *Bph3(t)*, *BPH32* and *BPH26* gene loci) gained virulence against related varieties with the same and different resistance genes, but planthoppers adapted to the resistant host IR65482-4-136-2-2 (*BPH10* locus) had reduced performance on phylogenetically distant plants with distinct resistant genes. In choice bioassays, avirulent planthoppers showed marked preferences for susceptible lines, whereas virulent planthoppers were less selective of varieties. We also examined whether virulence was associated with insecticide susceptibility. We tested susceptibility to three insecticides using a topical application method. Populations selectively reared on IR65482-4-136-2-2 had increased susceptibility to imidacloprid and fipronil, representing a possible trade-off with virulence. In contrast, a population with virulence to the highly resistant variety PTB33 was 4.88 × more resistant to imidacloprid and 3.18 × more resistant to BPMC compared to planthoppers of the same origin but reared on the susceptible variety IR22. Our results suggest complex relations between insecticide resistance and virulence that vary according to insecticidal toxins and resistance genes, and include potentially increased insecticide-susceptibility (a trade-off) as well as common detoxification mechanisms (a benefit).

## Highlights

• Costs and benefits of *Nilaparvata lugens* adaptation to resistant rice are assessed.
• *N. lugens* selected on IR65482-4-136-2-2 had reduced performance on other hosts.
• *N. lugens* selected on PTB33 and IR62 gained virulence against other resistant hosts.
• Virulence against IR65482-4-136-2-2 was associated with insecticide susceptibility.
• Virulence against PTB33 was associated with resistance to two insecticides.

## Graphical abstract

**Figure:**
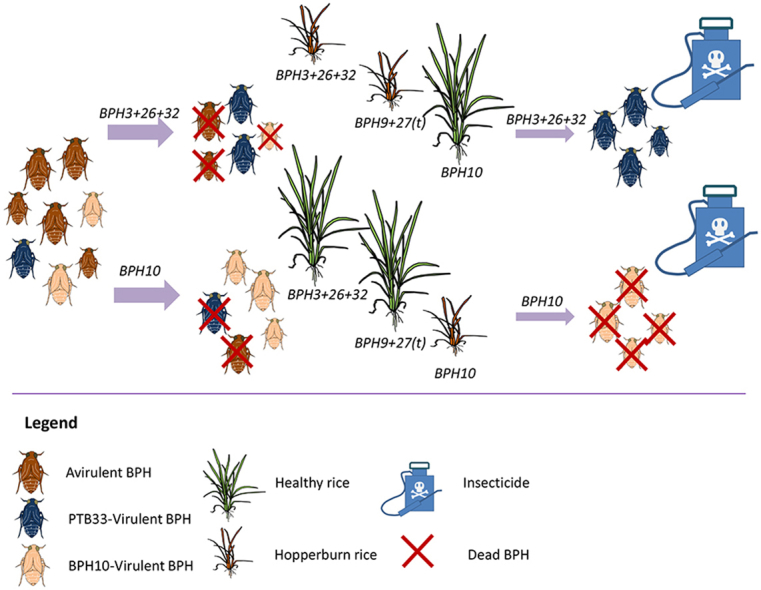

## 1 Introduction

The breeding of crop varieties with resistance to insect pests and pathogens has been the main focus of public research into pest management for the last several decades (Horgan, 2012, 2017). However, host plant resistance is limited by the often rapid development of virulent populations capable of damaging resistant crops. Among the best studied examples of adaptation for virulence is the Asian brown planthopper, *Nilaparvata lugens*, that developed virulence against rice, *Oryza sativa*, with the *Bph1* and *bph2* resistance genes during the 1980s and 1990s (Horgan, 2012; Fujita et al., 2013). Although virulence was first detected in localized populations (mainly in South East Asia), recent studies have indicated that adaptation by planthoppers to both these genes is now widespread (Myint et al., 2009; Horgan et al., 2017). These studies have also indicated that planthopper populations virulent against multiple resistance genes are now widespread throughout Asia, thereby severely restricting breeding programs and limiting the deployment of resistance genes in farmers’ fields. Furthermore, studies of migratory planthoppers suggest that virulence is often irreversible. For example, wild-collected, virulent planthoppers that have been successively reared on susceptible rice varieties over multiple generations can maintain virulence for at least several decades (Myint et al., 2009; Naeemullah et al., 2009; see also Kaneda and Kisimoto, 1979; Claridge and Den Hollander, 1982).

The maintenance of virulence among long-term populations of planthoppers implies that adaptation may be associated with ‘virulence genes’ that become fixed in small, caged populations. Recent studies have detected putative genes for virulence against the *Bph1* gene. However, different research groups have detected apparently different genetic sources of virulence to the same *Bph1* gene (Jing et al., 2014; Kobayashi et al., 2014). Other possible determinants of virulence adaptation include changes to the composition or functions of endosymbiotic bacterial and yeast communities involved in insect nutrition. A wide range of endosymbionts has been associated with feeding capacity in planthoppers and other phloem feeding insects (Douglas, 1998, 2009; Noda et al., 2012; Ferrater et al., 2013; Hansen and Moran, 2014; Horgan et al., 2019). Monitoring of endosymbionts during artificial selection for planthopper virulence has indicated shifts in the abundance of yeast-like symbionts (Lu et al., 2004; Chen et al., 2011a; Ferrater et al., 2015; Horgan and Ferrater, 2017) and in key functional bacteria (Tang et al., 2010; Wang et al., 2015; Xu et al., 2015).

The long-term maintenance of virulence in wild planthopper populations and caged colonies also implies that there are no fitness costs for adapted planthoppers, or that virulence bestows associated fitness benefits. Possible benefits include an extended intraspecific host range encompassing rice varieties or wild rice species with diverse genetic backgrounds and/or distinct resistance genes (Cheng, 1985; Ketipearachchi et al., 1998; Hirae et al., 2007; Horgan and Ferrater, 2017). Virulent planthoppers may also induce susceptibility of rice plants thereby enhancing the feeding success of virulent and avirulent conspecifics feeding together on both resistant and susceptible varieties (Ferrater and Horgan, 2016). Potential trade-offs against virulence adaptation may include increased susceptibility to natural enemies, reduced temperature tolerances, or increased susceptibilities to environmental toxins, including pesticides (Coustau et al., 2000). Studies have shown that avirulent planthoppers feeding on resistant hosts are often more susceptible to insecticides than planthoppers on susceptible hosts (reviewed in Horgan, 2018), but there is little information of the ecological effects on virulent planthoppers of insecticide applications to resistant crops (but see Chen et al., 2011b). Furthermore, resistance to insecticidal toxins such as pesticides, which is often associated with detoxification enzymes in insects or their endosymbionts (Dowd and Shen, 1990; Dowd, 1992; Zewen et al., 2003; Ding et al., 2013; Zhang et al., 2016), could result in cross-resistance with plant toxins, thereby reducing herbivore sensitivity to host plant defenses (Alyokhin and Chen, 2017).

In the present study, we examine possible fitness advantages and trade-offs associated with virulence in rice planthoppers. We reared planthoppers on susceptible and resistant rice plants for 20 + generations. During this time, populations had variously adapted to feed on the resistant varieties. We examined whether virulence predisposed planthoppers to feeding on rice varieties with similar resistance genes (i.e., genes located at the same or close positions along the rice genome), implying a fitness gain. We also examined whether virulent planthoppers had an increased (a fitness gain) or reduced (a trade-off) potential to feed on varieties with dissimilar genes or with distinct phylogenetic backgrounds. Finally, we examined whether virulence was associated with planthopper susceptibility or resistance to three commonly used insecticides. We suggest that a higher susceptibility of virulent populations would indicate a trade-off with virulence, but that higher insecticide resistance would indicate a common mechanism underlying the detoxification of chemicals and plant toxins because the plant defenses to which the planthoppers are exposed are less toxic than insecticides. To our knowledge, this is the first study to systematically examine intraspecific host range and insecticide susceptibility across a range of virulence-adapted herbivores. We discuss our results in the light of managing host plant resistance and virulence evolution in field crops.

## 2 Materials and methods

### 2.1 Plant materials

We used 21 rice lines during this study. Five lines (TN1, IR22, IR62, IR65482-4-136-2-2 and PTB33) were used as hosts for selection (henceforth natal hosts) of planthopper populations. These same lines, together with 16 further lines (henceforth 21 exposed hosts) were used to test feeding performance and examine virulence patterns. The lines included two susceptible controls (TN1 and IR22) and one tolerant variety (Triveni). The remaining lines have been associated with a range of planthopper resistant genes (Table S1); however, planthopper populations with virulence against these varieties are now widespread in Asia (Horgan et al., 2017). Aspects of regional virulence adaptation and phylogenetic relatedness among the varieties have been presented by Horgan et al. (2017). Seed was acquired from the International Rice Research Institute (IRRI). Traditional varieties were acquired through the International Network for Genetic Evaluation of Rice (INGER). The IR varieties were acquired through the Plant Breeding, Genetics and Biotechnology (PBGB) Division of IRRI.

### 2.2 Insect populations

We used ten planthopper populations in our experiments. In experiments 1 and 2 (see below) we used five colonies, each selected for over 20 generations on a different rice host. We used five South-Central Philippines Experimental Colonies (SCPEC, henceforth Region A) with TN1, IR22, IR62, IR65482-4-136-2-2, or PTB33 as natal hosts and four North Philippines Experimental Colonies (NPEC, henceforth Region B) with IR22, IR62, IR65482-4-136-2-2, or PTB33 as natal hosts. Production of the colonies, including selective rearing for 20 + generations and subsequent out-breeding, has been described in detail by Horgan and Ferrater (2017). A further colony, Laguna-TN1 was initiated with wild caught planthoppers from Pila (Laguna Province, Philippines) three years prior to our experiments. These planthoppers were continuously reared on TN1. Colonies were maintained in large aluminium wire mesh cages of 91.5 × 56.5 × 56.5 cm (H × L × W) under ambient light and temperature conditions in a greenhouse at IRRI. Only Region A colonies were used in Experiments 1 and 2, whereas the Region A, Region B and Laguna-TN1 colonies were used in Experiment 3. Because we required large numbers of planthoppers for our experiments, we divided our host-range bioassays into two experiments (1 and 2 below).

### 2.3 Experiment 1 – virulence of PTB33-selected colonies

**Fig. 1:**
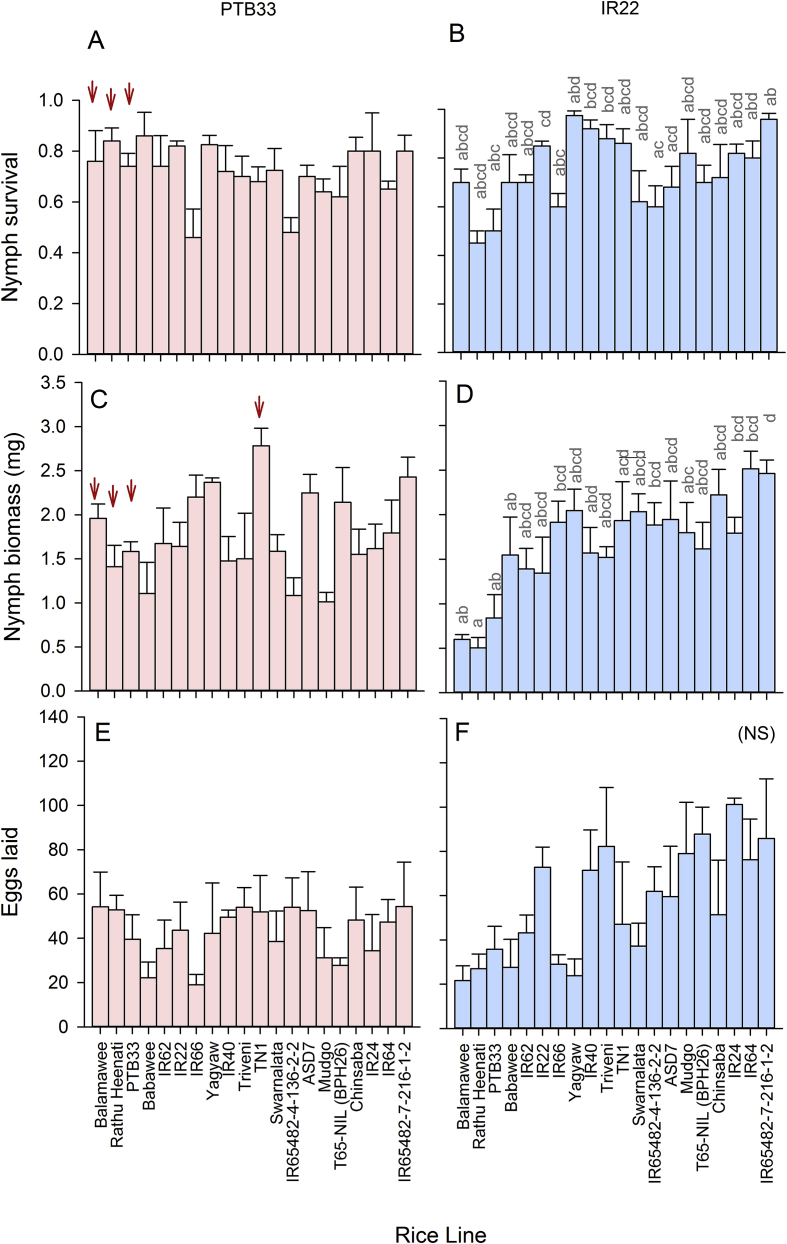
Fitness of *Nilaparvata lugens* from PTB33-selected (A,C,E) and IR22-selected (B,D,F) colonies on a range of susceptible and resistant rice varieties. Graphs indicate survival of nymphs (A,B), the biomass of survivors on whole plants (C,D), and the number of eggs laid per female (E,F) on each line. Standard errors are indicated; lowercase letters indicate homogenous variety groups (N = 5, Tukey ≥ 0.05); NS indicates no significant effect of exposed variety. Red, downward arrows indicate a decrease in variety resistance and increase in planthopper virulence (based on Duncan's many-to-one tests, P ≤ 0.05 and compared to IR22-selected colony). (For interpretation of the references to colour in this figure legend, the reader is referred to the Web version of this article.)

Planthoppers from PTB33-Region A and IR22-Region A (control) colonies were examined for performance on 20 rice lines (see Fig. 1, Table S1) using nymph survival and no-choice oviposition bioassays. The bioassays were conducted in a greenhouse at temperatures ranging from 25 to 37 °C as follows:

Nymph survival: To determine the performance of nymphs on each variety, 10 newly emerged nymphs were placed together on plants at 15 days after sowing (DAS). Plants were produced from pre-germinated seedlings in clay pots (7 × 11 cm; H × D) each enclosed in a cylindrical acetate cage (61 × 0.5 cm; H × D) with a mesh side window and top for ventilation. After 15 days, the survivors were collected and oven-dried at 60 °C for three days.

No-choice oviposition: The number of eggs laid on each variety was determined by confining two gravid female *N. lugens* on 15-day-old plants for 3 days. Plants were produced from seedlings in clay pots (7 × 11 cm; H × D) each enclosed in a cylindrical acetate cage (61 × 10.5 cm; H × D) with a mesh side window and top for ventilation. After three days, the insects were removed and the plants were collected and frozen at −20 °C. These plants were later dissected and the number of eggs laid on each plant was counted under a stereomicroscope (10 × magnification).

Bioassays were replicated five times in a completely randomized design. After each bioassay, the above-ground plant parts (shoots) were cut, placed in paper envelopes and oven-dried at 60 °C for at least 3 days before being weighed to estimate plant biomass.

### 2.4 Experiment 2 – virulence of IR62-and IR65482-4-136-2-2-selected colonies

**Fig. 2:**
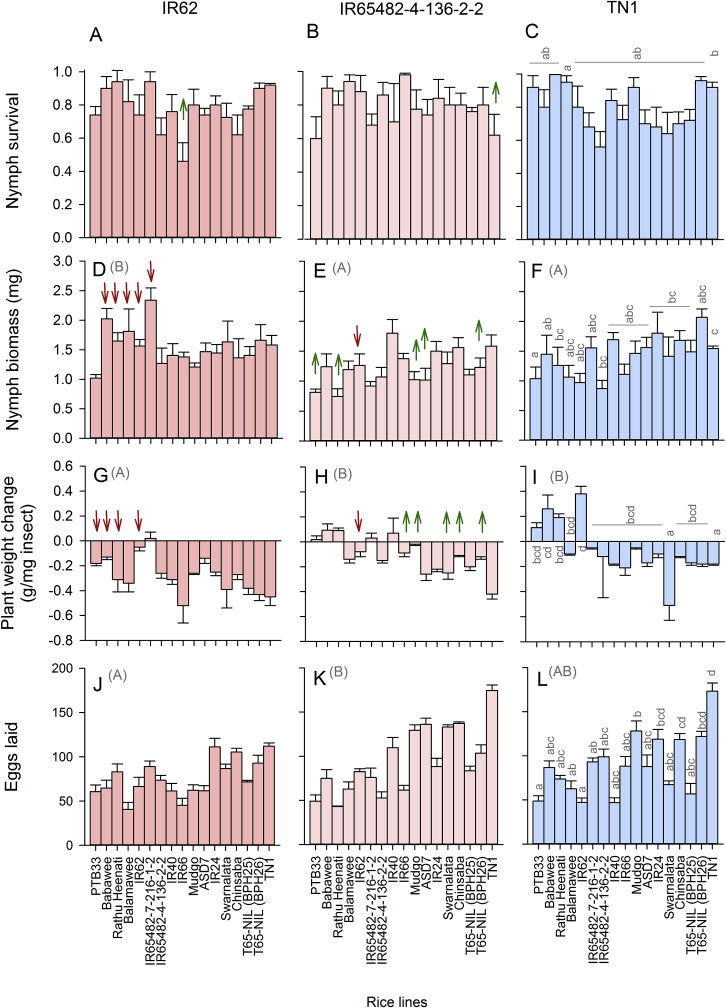
Fitness of *Nilaparvata lugens* from IR62-selected (A,D,G,J), IR65482-4-136-2-2-selected (B,E,H,K) and TN1-selected (C,F,I,L) colonies on a range of susceptible and resistant rice varieties. Graphs indicate the survival of nymphs (A–C) and biomass of nymphs on whole plants (D-F), plant weight change per unit planthopper weight relative to uninfested controls (G-I) and the number of eggs laid per female (J–L) on each line. Standard errors are indicated; lowercase letters indicate homogenous variety groups, uppercase letters in parentheses indicate homogenous colony groups (N = 5, Tukey ≥ 0.05). Red, downward arrows indicate a decrease in variety resistance (increased planthopper virulence) relative to TN1; green, upward arrows indicate an increase in resistance (decrease in virulence) (based on Duncan's many-to-one tests, P ≤ 0.05 and compared to TN1-selected colony). (For interpretation of the references to colour in this figure legend, the reader is referred to the Web version of this article.)

**Fig. 3:**
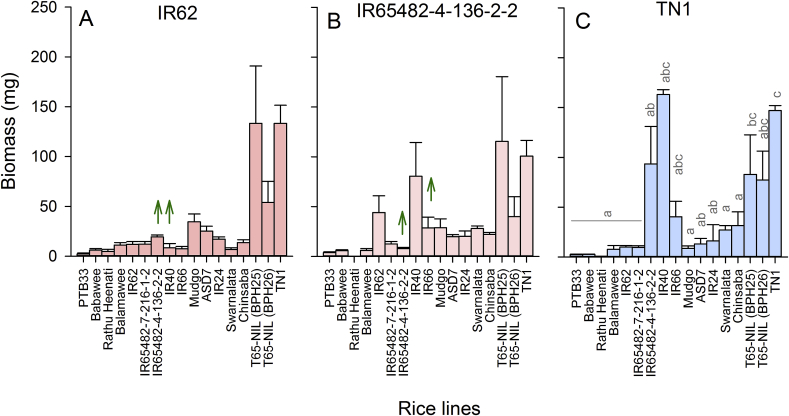
Results from biomass build-up experiment conducted with (A) IR62-selected, (B) IR65482-4-136-2-2-selected and (C) TN1-selected *Nilaparvata lugens* colonies. Standard errors are indicated; lowercase letters indicate homogenous variety groups (N = 5, Tukey ≥ 0.05). Green, upward arrows indicate an increase in resistance or decrease in planthopper virulence against mature rice plants (based on Duncan's many-to-one tests, P ≤ 0.05 and compared to TN1-selected colony). (For interpretation of the references to colour in this figure legend, the reader is referred to the Web version of this article.)

Planthoppers from IR62-Region A and IR65482-4-136-2-2-Region A colonies were examined for performance on 17 rice lines (see Fig. 2, Fig. 3, Table S1) and compared to planthoppers from the TN1-Region A (control) colony. Virulence was assessed using the nymph survival and no-choice oviposition bioassays (described above). During the nymph survival bioassays, a set of control plants (N = 5) was maintained to determine plant weight reduction due to planthopper feeding. We also conducted biomass build-up bioassays. The biomass build-up bioassays were conducted as follows:

Two gravid female *N. lugens* were confined on 30 DAS plants in pots (22 × 24 cm; H × D). The rice plants (and insects) were enclosed in organza cages (150 × 22 cm; H × D). The organza cloth was fitted around a cylindrical acetate-film base (30 × 22 cm; H × D) stably embedded in soil inside the pot and was supported by bamboo stakes and aluminium wire rings. The top, loose end of the cloth was tied to confine the insects. The females were left to lay eggs and the emerging nymphs were allowed to develop for 30 days. Planthoppers present in the cages after 30 days were collected using a mechanical aspirator and oven-dried during 3 days at 60 °C before being weighed.

The nymph survival and no-choice oviposition bioassays were carried out in a greenhouse at temperatures ranging from 25 to 37 °C. The biomass build-up bioassay was conducted in a screen-house facility with temperatures of 25–35 °C. All bioassays were replicated five times. After each bioassay, the above-ground plant parts (shoots) were cut, placed in paper envelopes and oven-dried at 60 °C for at least 3 days before being weighed to estimate plant biomass.

### 2.5 Experiment 3 – virulence and insecticide susceptibility

We examined the susceptibility of Region A and Region B colonies to three insecticides using a standard topical application method. At about the same time (within two weeks) that we monitored insecticide susceptibility, we also conducted choice settling and oviposition bioassays to check planthopper virulence. This was carried out because virulence strength can vary between generations, particularly after colony admixture (Ferrater et al., 2015; Horgan and Ferrater, 2017). Choice bioassays were conducted using plastic arenas (50 × 50 × 70 cm; L × W × H). The arenas each had five rice plants (20 DAS) in size-0 pots (7 × 11 cm; H × D), with one pot each for TN1, IR22, IR62, IR65482-4-136-2-2 and PTB33. Plants were arranged in a circular configuration taking care that the foliage did not touch other plants or the arena walls. To examine planthopper settling, 20 newly emerged nymphs were released to the centre of each cage. The positions of the nymphs (choice of plants) were noted after 48 h. To examine oviposition preferences, four gravid females were released to cages and allowed to oviposit. The females were removed after 3 days and plants dissected to count the eggs. Bioassays were each replicated five times (2 bioassays × 5 colonies × 5 replicates = 50 arenas).

Colonies that had been selected on IR22, IR62, IR65482-4-136-2-2 and PTB33 were screened for susceptibility to neonicotinoid [imidacloprid (99.9%)], phenylpyrazole [fipronil (97.9%)] and O-sec-butylphenyl methylcarbamate [BPMC (96.9%)] insecticides. Technical grade chemicals were supplied by Sigma Aldrich (Singapore). The colonies were compared against a wild population collected from Laguna (Philippines) and reared for ca 20 generations on TN1.

One to two-day old adult females were anaesthetized with carbon dioxide for about 10 s prior to the topical application of insecticides. A 0.2 μL droplet of insecticide in acetone solution was administered on the dorsal surface of the thorax of each individual insect using a Hamilton Repeating Dispenser (Hamilton Co., USA) adapted with a 10 μL microsyringe. The assays were conducted using six insecticidal concentrations and bioassays performed in triplicate using 20 individual insects per replication. The treated insects were maintained in transparent plastic boxes (5 × 10 cm, D × H) with rice seedlings (TN1) in an insectary at 27 °C and 12:12 h L:D. Insect mortality was recorded 24 h after treatment.

### 2.6 Data analyses

For experiments 1 and 2, we examined the response variables (eggs laid, nymph survival and growth [nymph biomass], plant weight reduction, and biomass build-up [population biomass]) for *N. lugens* on the range of rice varieties using univariate General Linear Models (GLM). The independent variables included in the models were ‘natal host’ (i.e., colony) and ‘exposed host’. Plant weight was initially included as a covariate in all models but had no significant effect and was subsequently removed. Tukey tests were used to determine homogeneous groups.

For each experiment, significant natal × exposed host interactions would indicate changes in the relative performance by the virulent colony on the different varieties compared to the avirulent colony. Where such changes were apparent, we examined data for shifts in performance relative to that on TN1 (ANOVAs with Duncan's post hoc tests). Settling by nymphs and oviposition by adults in the choice experiments were examined using univariate GLMs. Preferences were ranked by arena because of a lack of independence in choice experiments.

Probit analyses were conducted on insecticide susceptibility data using the PoloPlus© program (LeOra software, 2002) to estimate lethal doses – the concentrations required to kill 50% of the planthoppers (LD_50_s), the 95% fiducial limits, and slopes of the regression lines. Slopes were also examined for heterogeneity and parallelism. Colony LD_50_s were compared by considering non-overlapping fiducial limits as an indicator of significant differences (α = 0.05: Tabashnik et al., 1987). We ran a series of correlations between planthopper settling and oviposition on the natal hosts and corresponding LD_50_s for each insecticide. Residuals were examined after all analyses and found to be normal and homogeneous. With the exception of the Probit analyses, all other statistical analyses were conducted using SPSS version 23.0 (IBM SPSS Statistics).

## 3 Results

### 3.1 Experiment 1 – virulence of PTB33-selected colonies

Survival of PTB33-selected nymphs was higher than IR22-selected nymphs across varieties (natal host: F_1,165_ = 9.088, P < 0.005), this was mainly due to high survival of PTB33-selected nymphs on the resistant varieties Balamawee, Rathu Heenati and PTB33 (interaction; F_20,165_ = 2.915, P < 0.001: Fig. 1A and B). Althought there was a significant effect of exposed variety (exposed host; F_20,165_ = 4.035, P < 0.001: Fig. 1A and B), there were no differences between survival on the test varieties and TN1 (Fig. 1A and B). Similar trends were noted with nymph biomass; however, there was no difference between nymph biomass from the PTB33 or IR22-selected colonies (natal host: F_1,160_ = 0.922, P = 0.338). Nevertheless, the was a significant [natal host × exposed host] interaction indicating shifts in relative biomass (interaction: F_20,160_ = 2.443, P = 0.001). This interaction was due to a higher biomass of PTB33-selected nymphs on Balamawee, Rathu Heenati, PTB33 and TN1 (Fig. 1 A and 1B) compared to IR22-selected nymphs (indicating increased virulence of the adapted colonies on each of these lines). Biomass differed among varieties (exposed host: F_20,160_ = 4.327, P ≤ 0.001), but no variety was more resistant (lower biomass) than TN1 in the experiment (Duncan > 0.05: Fig. 1C and D).

Planthoppers from the two colonies laid similar numbers of eggs (F_1,160_ = 1.997, P = 0.160) and showed no differences in egg-laying across varieties (exposed host: F_19,160_ = 1.055, P = 0.402). There was no significant [natal × exposed host] interaction (interaction: F_19,160_ = 0.926, P = 0.552) (Fig. 1 E and 1F).

### 3.2 Experiment 2 – virulence of IR62- and IR65482-4-136-2-2-selected colonies

The was no effect of natal host on nymph survival among colonies derived from Region A (natal host: F_2,196_ = 0.550, P < 0.05: Fig. 2A–C). Nymph biomass in the survival bioassays was higher for colonies with IR62 as a natal host than colonies with either TN1 or IR65482-4-136-2-2 as natal hosts (natal host: F_2,196_ = 8.933, P < 0.001). Survival (exposed host: F_16,196_ = 32.506, P < 0.005) and biomass (exposed host: F_16,196_ = 3.631, P < 0.001) were affected by rice line with lower survival on Balamawee than TN1 and smaller nymphs developing on PTB33 and Babawee than on TN1 across all three natal hosts (Duncan's test > 0.05). There was a significant [natal host × exposed host] interaction for nymph survival (F_32,196_ = 2.045, P < 0.005) because of lower survival of nymphs reared on Mudgo from the IR62-selected colony and lower survival of nymphs on TN1 from the IR65482-4-136-2-2-selected colony. There was a significant [natal host × exposed host] interaction for nymph biomass (F_32,196_ = 1.692, P < 0.05) because of a higher biomass for IR62-selected nymphs on Babawee, Rathu Heenati, Balamawee, IR65482-7-216-1-2 and IR62 than for nymphs from the other colonies (Fig. 2D–F). Weight loss was greatest for plants attacked by IR62-selected nymphs (natal host: F_2,204_ = 7.018, P < 0.001) and was lower on PTB33, Babawee, Rathu Heenati and IR62 than on TN1 (exposed host: F16,204 = 5.159, P < 0.01; Tukey P ≤ 0.05). The interaction was significant (F_16,204_ = 1.682, P < 0.05) because of greater biomass losses to these latter varieties when attacked by IR62-selected planthoppers (Fig. 2 G). Compared to TN1-selected planthoppers, IR65482-4-136-2-2-selected planthoppers caused greater damage to TN1 (Fig. 2 H). Similar high damage was not apparent among the remaining varieties (Fig. 2 H).

IR62-selected planthoppers laid fewer eggs than planthoppers from the IR65482-4-136-2-2-selected colony (natal host: F_2,194_ = 14.653, P < 0.001) with most eggs laid on TN1, T65-NIL (*BPH26*), Chinsaba, and IR24 (exposed host: F_16,194_ = 4.025, P < 0.001). The interaction was not significant (Fig. 2J–L).

There was no colony effect (natal host: F_2,150_ = 0.755, P > 0.05) on biomass build-up in Experiment 2. TN1, T65-NILs and IR40 were most susceptible to planthopper damage (exposed host: F_16,150_ = 3.282, P ≤ 0.001: Fig. 2A–C). Although the interaction was not significant, IR62-selected planthoppers appeared less successful in feeding on IR65482-4-136-2-2 and IR40 than the TN1-selected colony, and the IR65482-4-136-2-2-selected colony was less successful in developing on T65-NIL (BPH26) and IR66 (Fig. 3A–C).

### 3.3 Experiment 3 – virulence and insecticide susceptibility

Choice settling and oviposition experiments indicated varying levels of adaptation to natal hosts between colonies at the time that we conducted insecticide susceptibility monitoring. Planthoppers reared on susceptible hosts showed significant preferences for settling on the same, or other susceptible hosts (Table 1). Planthoppers reared on resistant hosts were less selective during settling; however, in general, nymphs tended to settle predominantly on their natal hosts (except the Region A-PTB33-selected nymphs: Table 1). Similar trends were apparent from choice oviposition bioassays. Females reared on susceptible natal hosts laid more eggs on susceptible varieties, however the trend was statistically significant only for the Laguna (TN1) colony (Table 1). The results indicate that at the time of the experiments, the Region B colonies were better adapted to their resistant natal hosts than the Region A colonies, particularly the Region B-PTB33 planthoppers.

**Table 1:** Results of choice settling and oviposition bioassays conducted with *Nilaparvata lugens* colonies at the time of monitoring insecticide susceptibility.

Colony origin-natal host	Nymph settling (proportion of nymphs on each plant)						Oviposition (proportion of eggs on each plant)					
	Exposed host						Exposed host					
	TN1	IR22	IR65482-4-136-2-2	IR62	PTB33	F_4,20_	TN1	IR22	IR65482-4-136-2-2	IR62	PTB33	F_4,20_
Laguna-TN1	0.21 ± 0.06^a^	0.61 ± 0.09^b^	0.02 ± 0.02^a^	0.08 ± 0.03^a^	0.08 ± 0.02^a^	11.102***	0.20 ± 0.12^ab^	0.49 ± 0.10^b^	0.13 ± 0.16^ab^	0.13 ± 0.03^ab^	0.05 ± 0.04^a^	4.533**
Region A-IR22	0.19 ± 0.14^ab^	0.43 ± 0.17^b^	0.21 ± 0.13^ab^	0.17 ± 0.14^ab^	0.00 ± 0.00^a^	4.428**	0.13 ± 0.09	0.49 ± 0.18	0.14 ± 0.05	0.12 ± 0.07	0.11 ± 0.07	1.607
Region A-IR65482-4-136-2-2	0.14 ± 0.07	0.25 ± 0.08	0.29 ± 0.11	0.15 ± 0.08	0.17 ± 0.05	0.448	0.07 ± 0.05	0.41 ± 0.16	0.12 ± 0.05	0.19 ± 0.08	0.20 ± 0.08	0.908
Region A-IR62	0.06 ± 0.04	0.12 ± 0.10	0.26 ± 0.10	0.34 ± 0.10	0.22 ± 0.09	1.725	0.14 ± 0.10	0.28 ± 0.15	0.36 ± 0.11	0.13 ± 0.05	0.08 ± 0.03	1.419
Region A-PTB33	0.19 ± 0.12	0.31 ± 0.07	0.18 ± 0.10	0.22 ± 0.10	0.10 ± 0.04	1.525	0.34 ± 0.16	0.20 ± 0.10	0.26 ± 0.06	0.16 ± 0.05	0.04 ± 0.04	1.889
Region B-IR22	0.09 ± 0.07^a^	0.59 ± 0.08^b^	0.11 ± 0.07^a^	0.11 ± 0.06^a^	0.10 ± 0.10^a^	7.833***	0.28 ± 0.12	0.33 ± 0.16	0.26 ± 0.07	0.08 ± 0.06	0.04 ± 0.04	1.979
Region B-IR65482-4-136-2-2	0.22 ± 0.06	0.22 ± 0.06	0.33 ± 0.12	0.21 ± 0.08	0.02 ± 0.01	1.181	0.22 ± 0.16	0.24 ± 0.15	0.32 ± 0.20	0.10 ± 0.06	0.13 ± 0.06	0.381
Region B-IR62	0.16 ± 0.06	0.26 ± 0.09	0.15 ± 0.04	0.28 ± 0.10	0.15 ± 0.04	1.866	0.21 ± 0.08	0.26 ± 0.07	0.23 ± 0.06	0.10 ± 0.05	0.20 ± 0.12	1.108
Region B-PTB33	0.14 ± 0.05	0.16 ± 0.05	0.22 ± 0.07	0.16 ± 0.10	0.31 ± 0.11	1.026	0.23 ± 0.07	0.17 ± 0.08	0.28 ± 0.10	0.05 ± 0.03	0.27 ± 0.12	0.973

1: *** = P < 0.001, ** = P < 0.01, N = 5.

2: Lowercase letters indicate homogenous groups of exposed hosts.

The slopes of the insecticide-concentration/mortality relations were homogenous and parallel across colonies (Table 2). Compared to the Laguna colony, the Region A colonies had generally higher susceptibility to imidacloprid (0.201–0.888 versus 0.780–1.341 μg/g) and BPMC (13.536–46.007 versus 34.860–52.063 μg/g) and lower susceptibility to fipronil (0.288–1.865 versus 0.435–0.715 μg/g). Region B colonies, which included Laguna caught individuals as founders, also had lower susceptibility to fipronil (0.293–1.489 μg/g). This indicated consistent LD_50_ values within colony groups.

**Table 2:** Susceptibility of *Nilaparvata lugens* colonies to three insecticides.

Colony origin-natal host	Imidacloprid		Fipronil		BPMC	
	LD_50_ (μg/g) (95% FL)	Slope (SE)	LD_50_ (μg/g) (95% FL)	Slope (SE)	LD_50_ (μg/g) (95% FL)	Slope (SE)
Laguna-TN1	1.040 (0.780–1.341)	1.83 (0.24)	0.568 (0.435–0.715)	1.77 (0.22)	43.323 (34.860–52.063)	2.62 (0.31)
Region A-IR22	0.623 (0.389–0.888)	1.55 (0.29)	1.419 (1.081–1.865)^b^	2.00 (0.30)	35.938 (27.481–46.007)	2.20 (0.32)
Region A-IR65482-4-136-2-2	0.282 (0.201–0.382)*^,a^	1.95 (0.33)	1.052 (0.768–1.405)^b^	1.80 (0.29)	21.912 (15.520–28.661)^a^	2.03 (0.33)
Region A-IR62	0.455 (0.337–0.595)^a^	1.97 (0.30)	0.962 (0.716–1.233)^b^	2.56 (0.39)	21.518 (13.536–29.934)^a^	1.74 (0.32)
Region A-PTB33	0.424 (0.292–0.574)^a^	1.88 (0.32)	0.934 (0.288–1.853)	2.00 (0.33)	32.068 (22.173–43.385)	1.92 (0.32)
Region B-IR22	0.558 (0.364–0.779)^a^	1.86 (0.33)	1.053 (0.705–1.450)	1.90 (0.33)	36.990 (25.751–48.794)	2.51 (0.43)
Region B-IR65482-4-136-2-2	0.116 (0.083–0.154)*^,a^	2.09 (0.34)	0.399 (0.293–0.525)*	1.93 (0.30)	45.367 (30.046–60.980)	2.37 (0.42)
Region B-IR62	0.697 (0.485–0.955)	1.87 (0.32)	1.084 (0.793–1.421)^b^	1.94 (0.30)	38.547 (23.457–55.938)	1.67 (0.33)
Region B-PTB33	2.725 (1.722–3.884)**^,b^	1.83 (0.35)	1.106 (0.769–1.489)^b^	2.00 (0.33)	117.743 (74.394–165.546)**^,b^	1.65 (0.30)

* = significantly lower LD_50_ than colonies of same origin reared on a susceptible natal host.

** = significantly higher LD_50_ than colonies of same origin reared on a susceptible natal host.

a = significantly lower LD_50_ than the Laguna-TN1 population.

b = significantly higher LD_50_ than the Laguna-TN1 population.

Results indicated that the IR65482-4-136-2-2-selected colonies were more susceptible than corresponding IR22-selected colonies to imidicloprid (2 colonies) and fipronil (1 colony) (Table 2). The Region B PTB33-selected colony was less susceptible to imidacloprid and BPMC than the corresponding IR22-selected colony. The same was not observed among the Region A PTB33-selected planthoppers (Table 2). There were no significant correlations between settling or egg-laying in the choice bioassays and LD_50_ estimates.

## 4 Discussion

### 4.1 Intraspecific host range of virulent planthoppers

Artificial selection of planthoppers adapted to feed on resistant rice produced changes in key population traits. Among the more apparent changes were shifts in the capacity of populations to select, feed and oviposit on their natal hosts. However, this was accompanied by both physiological costs and benefits to the planthoppers. Among the benefits was a general improvement in feeding and egg-laying on other resistant rice lines. Planthoppers adapted to IR62 were better able to feed on Balamawee, Rathu Heenati, and PTB33 - a group of closely related traditional South Asian rice varieties (see phylogenetic analysis of varieties in Horgan et al., 2017). Rathu Heenati and PTB33 likely share resistance genes with IR62 (*Bph3(t)* and/or *Bph32*: Ren et al., 2016) and Balamawee contains the *Bph9* resistance gene (Nemoto et al., 1989). Planthoppers reared on IR62 had also adapted to a distantly related variety, Babawee, with the *bph4* gene that occurs at the same position as *Bph3* on rice chromosome 6 (Jairin et al., 2010) and may represent the same gene (Jairin et al., 2007). However, the IR62-reared planthoppers also demonstrated fitness reductions during selection – females laid fewer eggs than planthoppers with IR22 or IR65482-4-136-2-2 as natal hosts.

Planthoppers selected on PTB33 improved their relative ability to feed on closely related varieties (Balamawee and Rathu Heenati), but appeared less well adapted to IR62 that has similar genetic sources of resistance, and to Babawee. These virulent populations also had reduced fecundity, even on susceptible hosts. This occurred despite our efforts to reduce inbreeding by admixing populations from three sources into a single colony at post-selection. Despite the reduced fecundities, each of the populations that was reared on a resistant natal host had a broader intraspecific feeding range (encompassing more resistance genes) than planthoppers from susceptible natal hosts. This was particularly apparent in choice tests where, unlike planthoppers reared on a susceptible host, planthoppers from resistant natal hosts did not selectively settle or oviposit on IR22. Improved performance on a broader range of host varieties may also be associated with an increased feeding capacity of virulent planthoppers. For example, Ferrater and Horgan (2016) found that avirulent planthoppers gained more weight on rice plants (Triveni) that were previously attacked by IR62-selected planthoppers than on the same plants attacked by avirulent conspecifics. These authors suggested that virulent planthoppers had a greater capacity to induce host susceptibility and, based on controlled experiments, that virulent individuals transmitted unknown virulence factors to their feeding hosts.

The *Bph10* locus in IR65482-4-136-2-2 bestows only moderate resistance against the brown planthopper. Furthermore, it appears that once planthoppers have adapted to the gene (which occurs in <5 generations), the plants become highly favourable for nymph development and oviposition. Planthoppers adapted to *Bph10* and continually reared on IR65482-4-136-2-2 often attain higher body weights than planthoppers reared on highly susceptible varieties such as IR22 (Horgan and Ferrater, 2017, and this study). Furthermore, IR65482-4-136-2-2 plants are often preferred by planthoppers that have been artificially selected for virulence against other resistance genes (as noted in the oviposition choice bioassays in the present study). Because of low fecundities in IR62-selected and PTB33-selected planthoppers, but cross virulence to several resistant lines, we noted large shifts in planthopper rank performance associated with adaptation, such that virulent planthoppers performed relatively poorly on susceptible lines compared to wild populations or populations selected on susceptible natal hosts. For example, in our nymph survival bioassays, planthoppers selected on resistant natal hosts had apparently reduced virulence against several exposed susceptible lines (that contained a range of ineffective resistance genes). Our results therefore suggest that the rice-planthopper interactions include counterselection mechanisms that prevent selection of planthoppers virulent against one gene to adapt to certain other genes (Coustau et al., 2000). Based on our results, virulence against *Bph3/Bph32* and *Bph9* appears to be somewhat incompatible with virulence adaption against a range of other genes.

### 4.2 Virulence mechanisms

Several ideas have been put forward to explain virulence adaptation in herbivores (Douglas, 2009; Ferrater et al., 2013; Kobayashi, 2016). Using relict planthopper populations collected in Japan, Kobayashi et al. (2014) suggested that a single recessive gene governs virulence against *Bph1* in planthoppers. At about the same time, using similar experimental populations, Jing et al. (2014) identified a virulence locus *Qhp7* (chromosome 7) that governs preference for plants with the *Bph1* gene, and two major QTLs (*Qgr5* and *Qgr14:* chromosome 5) that govern rates of insect growth on resistant plants. Despite the planthoppers in both studies having virulence against the *Bph1* gene (derived from the variety ‘mudgo’), the genetic mechanisms did not concur. This suggests that there may be some redundancy in virulence genes with several different genes bestowing virulence against the same resistance gene.

In recent years, considerable research attention has focused on the role of endosymbiotic microorganisms in virulence adaptation (Douglas, 1998, 2009; Noda et al., 2012; Ferrater et al., 2013; Hansen and Moran, 2014; Horgan et al., 2019). A number of studies have found that the densities of YLS in planthoppers vary depending on the nature of the plant host (vis-a-vis resistant or susceptible) and that densities change during selection for virulence. However, the results from similar studies have not been consistent regarding the direction of change, with authors suggesting that densities are both lower (Ferrater et al., 2015; Horgan and Ferrater, 2017) and higher (Lu et al., 2004; Chen et al., 2011a) in planthoppers on resistant hosts. Similarly, results from studies that examined differences in bacterial sysmbionts from the same virulent and avirulent planthopper populations have given inconsistent results (Tang et al., 2010; Wang et al., 2015; Xu et al., 2015). More recently, a study with green leafhoppers, *Nephotettix virescens*, has indicated consistent relations between abundance of the obligate symbiont *Candida sulcus* and virulence patterns following artificial selection (Horgan et al., 2019). A potential for endosymbiotic bacteria to determine planthopper virulence is noteworthy because of the role of bacteria in adaptation by insect herbivores to insecticidal proteins (Dowd and Shen, 1990; Dowd, 1992). As a final note on mechanisms, it is likely that the planthoppers, or their symbionts may undergo epigenetic shifts towards greater tolerance of resistant rice. This hypothesis is currently difficult to test because of persistent gaps in knowledge of rice resistance mechanisms and of virulence adaptation mechanisms – including information on the specific genes involved in both (Ferrater et al., 2013; Horgan, 2018).

### 4.3 Insecticide resistance

Evolutionary pressures on herbivorous insects has resulted in species evolving a range of behavioural and physiological mechanisms to overcome the host's toxic secondary chemicals, antifeeding and antidigestion proteins, repellent volatiles and/or nutrient deficiencies (Coustau et al., 2000; Ferrater et al., 2013; Alyokhin and Chen, 2017). Furthermore, because of the diversity of defensive compounds that plants possess, herbivores, particularly specialist phloem-feeders (Alyokhin and Chen, 2017) are equipped with multiple detoxification mechanisms. These allow herbivores to also deal with other environmental toxins including xenobiotics such as insecticides. Continuous exposure to specific insecticides could therefore increase the production of general insect detoxification enzymes leading to cross-resistance against different insecticides (e.g., Tang et al., 2010; Matsumura et al., 2014), and potentially cross-resistance between insecticides and plant-derived insecticidal toxins. This could potentially occur where insecticide resistance is associated with constitutive overexpression of detoxifying enzymes in the herbivore. For example, cytochrome P450s have been associated with both insecticide resistance and virulence adaptation in planthoppers and other herbivores (Coustau et al., 2000; Zimmer et al., 2018). Endosymbiotic bacteria could also possess such detoxification mechanisms, which may be rapidly activated through bacterial multiplication in the insect mutualist (Dowd, 1992). Planthoppers possess a number of genes, including redundant genes, for a diversity of cytochrome P450s. The overexpression of certain of these P450 genes has been associated with resistance to imidacloprid and fipronil in field populations of planthoppers (Zewen et al., 2003; Tang et al., 2010; Ding et al., 2013; Zhang et al., 2016). Other mechanisms, including target site insensitivity may also be involved, and can lead to irreversible resistance (Tang et al., 2010; Ding et al., 2013).

Despite the common detoxification methods related to both insecticides and plant chemicals, few studies have examined the relationship between insecticide resistance and virulence. Chen et al. (2011b) examined the fitness costs of imidacloprid-resistance in brown planthoppers from China. These authors found than planthoppers from an insecticide-resistant population had lower population growth rates compared to those from an insecticide-susceptible population. However, the authors also indicated that the relative fitness of insecticide-resistant planthoppers was higher than that of insecticide-susceptible planthoppers when reared on the resistant variety IR36 (*bph2*). In a study by Yang et al. (2014), insecticide-resistant planthoppers continually reared on TN1 in closed colonies, became increasingly susceptible to imidacloprid and chlorpyrifos over 14 generations. As the planthoppers lost resistance, they became increasingly less virulent against IR26 (*Bph1*-gene) and IR36 (*bph2*-gene), but also had greater fitness on the susceptible natal host (TN1). In our experiments, of two populations from the PTB33 natal host, one showed high virulence (noted in choice bioassays) at the time of our insecticide susceptibility bioassays. The second population, although it had improved performance on resistant hosts (e.g., was less selective of IR22), was apparently not yet well adapted to PTB33. Our monitoring of the development of virulence in these colonies (published in Ferrater et al., 2015) indicated that during the artificial selection of planthoppers on PTB33, population sizes were unstable and virulence fluctuated between generations. At the time we conducted experiment 3, only the selected Region B colony had significant virulence against PTB33. Therefore, our results suggested a high concordance between confirmed virulence against PTB33 and a low susceptibility to imidacloprid and BPMC (insecticide resistance). This was further highlighted by relatively high insecticide susceptibilities in the remaining Region B populations – each of which had similar population origins to the PTB33-selected colony.

Our results suggest that by selecting for virulent planthoppers on PTB33, we simultaneously selected for insecticide resistance. What is less clear is whether insecticide resistance was a cause or consequence of virulence selection. Because PTB33 is less toxic than insecticides, we suggest that insecticide resistance is most probably the cause of virulence in this case. This can be explained through the current understanding of virulence-selection processes in planthoppers (see Horgan, 2018). It is now increasingly apparent that wild planthopper populations include rare individuals with virulence against specific resistance genes. These ‘forerunners’ are thought to be selected during successive exposure to resistant plants thereby shifting the population demography toward domination by virulent individuals (the descendants of previously rare forerunners). We suggest that in our experiment with Region B populations selected on PTB33, these forerunners were apparently also resistant to two synthetic toxins (Table 2). Exposure to pesticides (particularly imidacloprid) may have resulted in a site mutation or constitutive overexpression of P450s (in the herbivore or bacteria) resulting in insecticide-resistant individuals that were also capable of detoxifying plant toxins from PTB33. Such a mechanism, where the overuse of pesticides primes populations for virulence to relatively less toxic plant defenses, could explain why planthopper virulence has been detected against many resistant rice varieties/genes that have never been widely exposed to field populations of planthoppers (Myint et al., 2009; Horgan et al., 2017). Our results are convincing because our virulent and avirulent (IR22) populations were selected from the same founder populations. Further research is required to verify such an association in planthoppers and other herbivores and to determine potential underlying physiological and genetic mechanisms.

In contrast to the apparent association between insecticide resistance and virulence against PTB33 in our Region B colony, we found a potential trade-off between insecticide susceptibility and virulence against the *Bph10* gene. In our study, two populations selected on IR65482-4-136-2-2 were more susceptible to imidacloprid than populations with the same origins but reared on a susceptible variety. Furthermore, one of the colonies was also more susceptible to fipronil than avirulent planthoppers from the same origin. Such trade-offs may be related to a greater feeding capacity, larger size or more active planthoppers from the IR65482-4-136-2-2 natal host. Differences between the reactions of PTB33-selected and IR65482-4-136-2-2-selected planthoppers to the same insecticides in our study indicate complex relations between virulence and insecticide resistance that probably depend on the specific rice resistance or planthopper virulence genes, as well as the chemical nature and mode of action of the specific insecticide.

Our results indicate that to achieve efficient management of herbivore virulence, greater attentionshould be placed on deploying genes that are counterselective for virulence either in pyramided or monogenic lines, and on the compatibility of host plant resistance with regional trends in agrochemical use.
