# Supplementary material for: Changes in insecticide resistance and host range performance of planthoppers artificially selected to feed on resistant rice
Source: Crop Prot. 2020 Jan;127:104963. doi: 10.1016/j.cropro.2019.104963 (PMC6894310; doi:10.1016/j.cropro.2019.104963)
Supplement: Multimedia component 1 [file mmc1.doc]

Supplementary Table S1. Twenty-one rice lines used in experiments with *Nilaparvata lugens*, indicating origins and putative resistance genes or quantitative trait loci (QTLs)

| Varieties | Cluster^1^ | Origin and development | Putative genes | References |
| --- | --- | --- | --- | --- |
| Balamawee | C2b-1 | Traditional variety, Sri Lanka | *Bph9, Bph27(t)* | Nemoto et al., 1989; He et al. 2013 |
| PTB33 | C2b-1 | Traditional variety, India | *Bph32, Bph3(t), BPH26* | Ren et al., 2016 |
| Rathu Heenati | C2b-1 | Traditional variety, Sri Lanka | *Bph3, Bph17* | Sun et al., 2005; Jairin et al., 2007 |
| IR22 | C2b-2 | Released by IRRI in 1978 (Philippines) | None | Khush and Virk, 2005 |
| IR24 | C2b-2 | Released by IRRI in 1978 (Philippines) | *Bph1* | Khush and Virk, 2005 |
| IR40 | C2b-2 | Released by IRRI in 1977 (Philippines) | *bph2* | Khush and Virk, 2005 |
| IR62 | C2b-2 | Released by IRRI in 1984 (Philippines) | *Bph3(t) or Bph32^3^* | Ren et al., 2016 and Horgan et al., 2017 |
| IR64 | C2b-2 | Released by IRRI in 1985 (Philippines) | *Bph1* | Khush and Virk, 2005 |
| IR65482-4-136-2-2 | C2b-2 | Breeder’s line developed through introgression with *Oryza australiensis* Domin | *Bph10* | Ishii et al., 1994 |
| IR65482-7-216-1-2 | C2b-2 | Breeder’s line developed through introgression with *Oryza australiensis* Domin | *Bph18* | Jena et al., 2006 |
| IR66 | C2b-2 | Released by IRRI in 1987 (Philippines) | *bph4* | Khush and Virk, 2005 |
| Swarnalata^2^ | C2b-2 | Traditional variety, Bangladesh | *Bph6* | Nemoto et al., 1989 |
| Yagyaw | C2b-2 | Traditional variety, Vietnam | *Qbph3, Qbph9* | Liu et al., 2009 |
| T65-NIL (*BPH25)* | C2b-3^2^ | Near-isogenic line developed at Kyushu University, Japan, with donor ADR52 (India) and recurrent parent T65 | *BPH25* | Yara et al., 2010 |
| T65-NIL (*BPH26)* | C2b-3^2^ | Near-isogenic line developed at Kyushu University, Japan, with donor ADR52 (India) and recurrent parent T65 | *BPH26* | Yara et al., 2010 |
| Taichung Native 1 (TN1) | C2b-3 | Released in Taiwan in 1960 | None | De Datta, 1981 |
| ASD7 | C2b-4 | Traditional variety, India | *bph2, qBPH6, qBPH12* | Athwal et al., 1971; Mai et al., 2015 |
| Babawee | N | Traditional variety, Sri Lanka | *bph4* | Lakshminarayana and Khush, 1977; Jairin et al., 2010 |
| Chinsaba | N | Traditional variety, Myanmar | *bph8* | Nemoto et al., 1989 |
| Mudgo | N | Traditional variety, India | *Bph1* | Kim and Sohn, 2005; Sidhu et al., 2005 |
| Triveni | N | Traditional variety, India | None (tolerance) | Ho et al., 1982 |

1: Clusters indicate relatedness according to results of Single Nucleotide Polymorphism analysis presented in Horgan et al. (2017)

2: Based on recurrent parent T65

3: Genes present in IR62 and derived from PTB33 have not been determined but are thought not to include *BPH26* (a synonym for *bph2*).

**References for Table S1**

Athwal, D., Pathak, M., Bacalangco, E., Pura, C., 1971., Genetics of resistance to brown planthoppers and green leafhoppers in *Oryza sativa* L. Crop Sci. 11, 747-750.

De Datta, S.K., 1981. Principles and practices of rice production. International Rice Research Institute, Los Baños (Philippines).

He, J., Liu, Y., Liu, Y., Jiang, L., Kang, H., Liu, S., Chen, L., Liu, X., Cheng, X., Wan, J., 2013. High-resolution mapping of brown planthopper (BPH) resistance gene *Bph27(t)* in rice (*Oryza sativa* L.) Mol. Breed. 31, 549-557.

Ho, D., Heinrichs, E., Medrano, F., 1982. Tolerance of the rice variety Triveni to the brown planthopper, *Nilaparvata lugens*. Environ. Entomol. 11, 598-602.

Horgan, F.G., Srinivasan, T.S., Bentur, J.S., Kumar, R., Bhanu, K.V., Sarao, P.S., Chien, H.V., Almazan, M.L.P., Bernal, C.C., Ramal, A.F., Ferrater, J.B., Huang, S.-H., 2017. Geographic and research center origins of rice resistance to Asian planthoppers and leafhoppers: implications for rice breeding and gene deployment. Agronomy. 7, 62.

Ishii, T., Brar, D., Multani, D., Khush, G., 1994. Molecular tagging of genes for brown planthopper resistance and earliness introgressed from *Oryza australiensis* into cultivated rice, *O. sativa*. Genome. 37, 217-221.

Jairin, J., Phengrat, K., Teangdeerith, S., Vanavichit, A., Toojinda, T., 2007. Mapping of a broad-spectrum brown planthopper resistance gene, *Bph3*, on rice chromosome 6. Mol. Breed. 19, 35-44.

Jairin, J., Sansen, K., Wongboon, W., Kothcharerk, J., 2010. Detection of a brown planthopper resistance gene *bph4* at the same chromosomal position of *Bph3* using two different genetic backgrounds of rice. Breed. Sci. 60, 71-75.

Jena, K., Jeung, J., Lee, J., Choi, H., Brar, D., 2006. High-resolution mapping of a new brown planthopper (BPH) resistance gene, *Bph18* (t), and marker-assisted selection for BPH resistance in rice (*Oryza sativa* L.). Theor. Appl. Genet. 112, 288-297.

Khush, G., Virk, P., 2005. IR Varieties and their impact. International Rice Research Institute, Los Baños (Philippines).

Kim, S.-M., Sohn, J.-K., 2005. Identification of a rice gene (*Bph 1*) conferring resistance to brown planthopper (*Nilaparvata lugens* Stål) using STS markers. Mol. Cells. 20, 30-34.

Laksminarayana, A., Khush, G.S., 1977. New genes for resistance to the brown planthopper in rice. Crop Sci. 17, 96-100.

Liu, Y., Su, C., Jiang, L., He, J., Wu, H., Peng, C., Wan, J., 2009. The distribution and identification of brown planthopper resistance genes in rice. Hereditas. 146, 67-73.

Mai, T.V., Fujita, D., Matsumura, M., Yoshimura, A., Yasui, H., 2015. Genetic basis of multiple resistance to the Brown planthopper (*Nilaparvata lugens* Stål) and the green rice leafhopper (*Nephotettix cincticeps* Uhler) in the rice cultivar ‘ASD7’ (*Oryza sativa* L. ssp. *indica*). Breed. Sci. 65, 420-429.

Nemoto, H., Ikeda, R., Kaneda, C., 1989. New genes for resistance to brown planthopper, Nilaparvata lugens Stal, in rice. Jpn. J. Breed. 39, 23-28.

Ren, J., Gao, F., Wu, X., Lu, X., Zeng, L., Lv, J., Su, X., Luo, H., Ren, G., 2016. *Bph32*, a novel gene encoding an unknown SCR domain-containing protein, confers resistance against the brown planthopper in rice. Sci. Rep. 6, 37645.

Sidhu, N., Bansal, U., Shukla, K., Saini, R., 2005. Genetics of resistance to whitebacked planthopper in five rice stocks. Genetics 37, 1-11.

Sun, L., Su, C., Wang, C., Zhai, H., Wan, J., 2005. Mapping of a major resistance gene to the brown planthopper in the rice cultivar Rathu Heenati. Breed. Sci. 55, 391-396.

Yara, A., Phi, C.N., Matsumura, M., Yoshimura, A., Yasui, H.. 2010. Development of near-isogenic lines for BPH25 (t) and BPH26 (t), which confer resistance to the brown planthopper, Nilaparvata lugens (Stål.) in indica rice ‘ADR52’. Breed. Sci. 60, 639-647.
